# Supplementary material for: Data-driven estimation of the instantaneous reproduction number and growth rates for the 2022 monkeypox outbreak in Europe
Source: PLoS One. 2023 Sep 13;18(9):e0290387. doi: 10.1371/journal.pone.0290387 (PMC10499224; doi:10.1371/journal.pone.0290387)
Supplement: S1 File — (PDF) [file pone.0290387.s001.pdf]

Supplementary Material: Data-driven estimation of the instantaneous reproduction number and growth rates for the 2022 monkeypox outbreak in Europe

Fernando Saldaña[1,*], Maria L. Daza-Torres[2], Maíra Aguiar[1,3,4],

1 Basque Center for Applied Mathematics (BCAM), Bilbao, Spain
2 Department of Public Health Sciences, University of California Davis, California, United States
3 Ikerbasque, Basque Foundation for Science, Bilbao, Spain
4 Dipartimento di Matematica, Università degli Studi di Trento, Trento, Italy

* fsaldana@bcamath.org

List of Supplementary Materials

S1 Prior distributions density
Figs S1-S7
S2 Synthetic Examples

S1 Prior distributions density

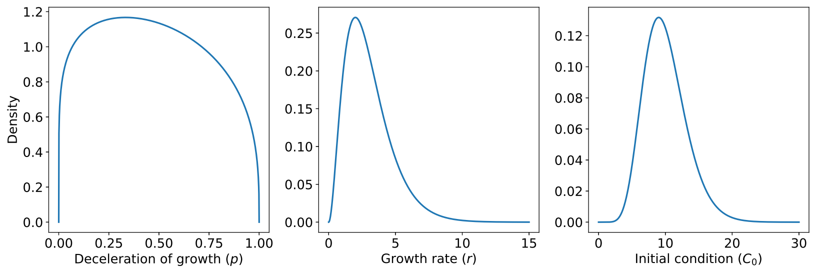

Fig S1. Density of the prior distribution in the adaptive sequential Bayesian approach for the parameters: deceleration of growth (p), growth rate (r), and initial condition (C_0).

S2 Synthetic Examples

To assess the accuracy of our parameter recovery process, we utilize a synthetic data set generated by the observational model (Equation 6, main file). Our objective is to estimate the values of the parameters r, p, and C_0. We employ Bayesian estimation by training the model with only one data window and leveraging it to predict the observed cases. Furthermore, we present a comparative analysis by contrasting the outcomes obtained from the Bayesian sequential approach.

Example 1

In this scenario, parameters r and p are constant throughout the observed period. The synthetic data was generated using the following values: $r = 10$, $p = 0.4$, $C_0 = 20$, $\theta = 0.01$ and $\omega = 2$. Figure S2 displays the resulting synthetic data.

We employ the Bayesian approach with a dataset consisting of 35 data points to train the model and make predictions. We aim to predict the next 65 days. Since the parameters r and p remain constant over the entire time interval, training the model with the initial dataset is sufficient to make accurate predictions. In this case, using the adaptive approach does not yield any additional information, as the underlying dynamics remain the same. You can observe the posterior distribution in Figures S3 and S4. The training set is represented by the gray region, while the blue region indicates the forecasted period. The solid red lines depict the median incidence forecast. The darker-shaded blue region represents the interquartile forecast range, indicating the middle 50% of the predicted values. The lighter-shaded blue region indicates the 5th to 95th percentile range, encompassing a wider range of possible outcomes. The observed cases are denoted as individual data points.

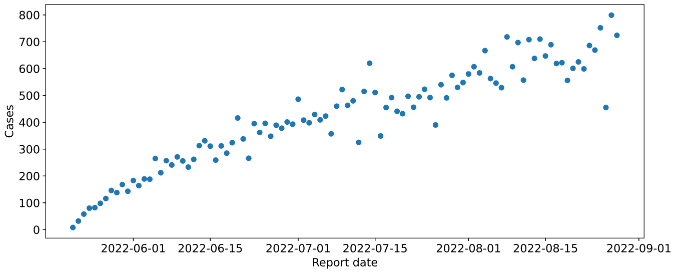

Fig S2. Synthetic data generated with the Negative Binomial model (Equation 6, main file) with parameters $r = 10$, $p = 0.4$, $C_0 = 20$, $\theta = 0.01$ and $\omega = 2$.

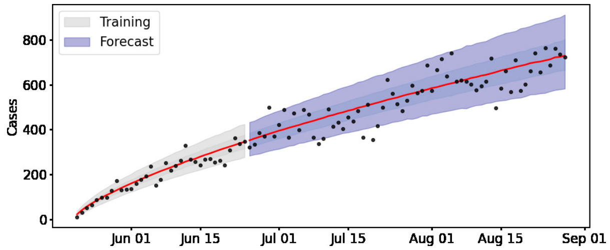

Fig S3. Forecasting incidence time series. The training set is represented by the gray region, while the blue region indicates the forecasted period. The solid red lines depict the median incidence forecast. The darker-shaded blue region represents the interquartile forecast range, indicating the middle 50% of the predicted values. The lighter-shaded blue region indicates the 5th to 95th percentile range. The observed cases are denoted as individual data points.

Example 2

We generate a synthetic data set assuming that the values of r, p, and C_0 change over time. We utilized simple piecewise functions. Specifically, we defined $r(t) = R[k]$ and

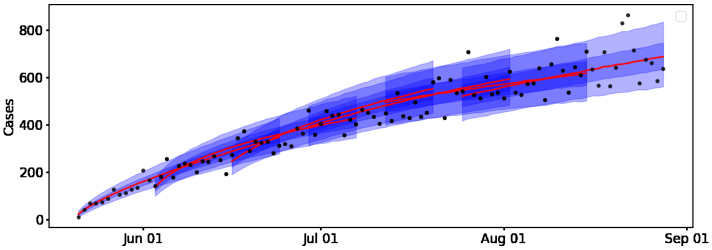

Fig S4. The predicted incidence time series is generated using the sequential Bayesian approach method with a window size of 35 data points and moving every 14 days. The solid red lines depict the median incidence forecast. The darker-shaded blue region represents the interquartile forecast range, indicating the middle 50% of the predicted values. The lighter-shaded blue region indicates the 5th to 95th percentile range. The observed cases are denoted as individual data points.

$p(t) = P[k]$ for $t \in [t_{nk}, t_{(n+1)k}]$, where $k = 0, \ldots 4$, $n = 20$, $R = [5, 6, 8, 9, 10]$ and $P = [0.2, 0.25, 0.35, 0.45, 0.5]$.

We initialized the variable C_0 as 20 and applied the GGM dynamic to update its value. Additionally, we kept the parameters θ and ω fixed at 0.04 and 3, respectively. The synthetic data generated from these settings are visualized in Figure S5.

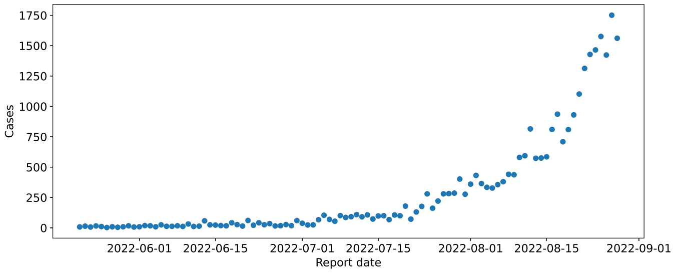

Fig S5. Synthetic data generated with the Negative Binomial model (Equation 6, main file) with fixed dispersion parameters $\theta = 0.04$ and $\omega = 3$ and time-dependent parameters for r, p.

We utilized two models in our analysis: the Bayesian sequential model and a Bayesian model assuming constant parameters for r and p. The Bayesian sequential model involved a window size of 35 days, with updates every 14 days. The Bayesian model that assumed constant parameters utilized 35 data points for training, similar to Example 1.

However, it is crucial to highlight that the estimation obtained from the model assuming constant parameters and a fixed-size window was poor. This can be attributed to the fact that the model was trained on a dataset with low counts observed at the beginning of the outbreak while assuming constant values for r and p. Consequently, the predictions made by this model were relatively flat. Please refer to Figure S6 for a visual representation.

On the other hand, by acknowledging that the parameters r and p can change over time and adopting the sequential Bayesian approach, we were able to capture these variations and achieve improved predictive performance. The sequential Bayesian approach adjusts to the changing parameters, making more accurate predictions. Figure

S7 provides a visual depiction of the enhanced performance achieved through this approach.

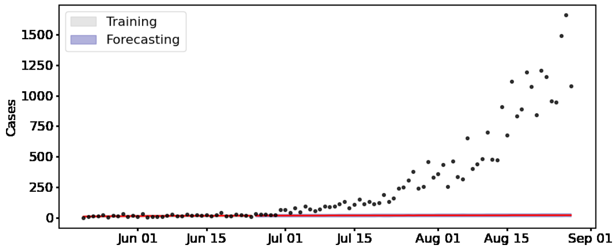

Fig S6. The predicted incidence time series is illustrated in the plot. The training set is represented by the gray region, while the blue region indicates the forecasted period. The solid red lines depict the median incidence forecast. The darker-shaded blue region represents the interquartile forecast range, indicating the middle 50% of the predicted values. The lighter-shaded blue region indicates the 5th to 95th percentile range.

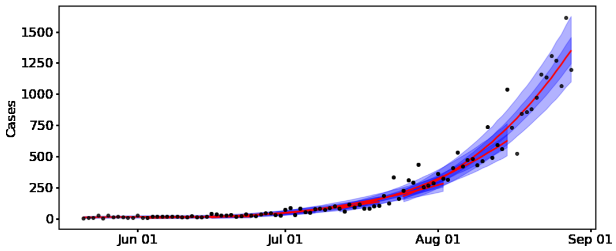

Fig S7. The predicted incidence time series is generated using the sequential Bayesian approach method with a window size of 35 data points and moving every 14 days. The solid red lines depict the median incidence forecast. The darker-shaded blue region represents the interquartile forecast range, indicating the middle 50% of the predicted values. The lighter-shaded blue region indicates the 5th to 95th percentile range.
