# Supplementary material for: Data-driven estimation of the instantaneous reproduction number and growth rates for the 2022 monkeypox outbreak in Europe
Source: PLoS One. 2023 Sep 13;18(9):e0290387. doi: 10.1371/journal.pone.0290387 (PMC10499224; doi:10.1371/journal.pone.0290387)
Supplement: Attachment — Submitted filename: ResponseReviewers.pdf [file pone.0290387.s002.pdf]

basque center for applied mathematics

Basque Center for Applied Mathematics - BCAM
Alameda de Mazarredo 14
Bilbao, Bizkia, Spain
Zipcode: 48009
E-mail: fsaldana@bcamath.org

July 20, 2023

Dear reviewers,

We wish to extend our appreciation for your work and constructive feedback. My co-authors and I have carefully attended the comments. We have modified the manuscript accordingly (the changes are marked in red). Our responses are given in a point-by-point manner below.

Sincerely,

Fernando Saldaña

Reviewer 1. This is an interesting study reporting estimates of the reproduction number of the monkeypox epidemics in several European countries. I enjoyed reading the paper, and only have a few clarifying comments:

1) Did authors employ the 10-day smoothed daily time series or the raw time series? Initially I thought they had employed the smoothed series presented in figure 1. However, authors accounted for the overdispersion in the observation model when fitted the models to the data. Is it necessary to characterize overdispersion if the smoothed time series are used?

1) Response. Yes, we utilized the 10-day smoothed daily time series in our model. This smoothing approach was implemented to mitigate reporting delays and address anomalies in the monkeypox incidence data, enhancing the quality of the time series. The moving average played a crucial role in our analysis by reducing noise and highlighting underlying trends or patterns.
However, it is important to note that smoothing techniques, such as the moving average, do not inherently address the issue of overdispersion commonly observed in epidemiological data. Overdispersion arises when the observed variance in the data exceeds what would be expected from theoretical distributions like the binomial or negative binomial distribution.
Given the prevalence of overdispersion in epidemiological data, it remains necessary to characterize and account for this phenomenon even when using a smoothed time series. Relying solely on smoothing techniques may not fully capture or account for the excessive variation inherent in the data. To address this concern, we incorporated a negative binomial model in our analysis, which explicitly accounts for overdispersion. We have included a dedicated paragraph in the revised paper to clarify this matter. (See Page 6, Lines 168-179)

2) Authors employed a Gamma (25, 0.5) distribution to model the generation time distribution of monkeypox. Did authors obtain this distribution directly from the Gozzueta et al EID article? Just to be sure that the authors are using the same distribution inferred in the Gozzueta paper.

2) Response. In the paper by Gozzueta et al. the authors only mentioned that the mean generation time for the current mpox outbreak in Italy was estimated to be 12.5 days (95% CI of the mean: 7.5-17.3; 5th and 95th percentiles of the distribution 5–23 days). In the main text, Gozzueta et al. only mentioned this estimation but they did not mention the specific values for the generation time distribution. Hence, we proposed the $Gamma(25, 0.5)$ (see

the blue plot below) as an attempt to mimic the estimation found by Gozzueta. However, checking the online supplementary material in Gozzueta et al. we now found that the exact distribution inferred by Gozzueta et al. is a $Gamma(4.85, 2.27)$ (see the red plot below). Although we think our proposed $Gamma(25, 0.5)$ was appropriate to approximate the generation time distribution described in the main text of Gozzueta, we have now adopted the $Gamma(4.85, 2.27)$ inferred by Gozzueta. This new distribution affects the estimation of R_t. However, the quantitative change in the estimated R_t is relatively small and does not affect any of the conclusions of this work. The changes in the estimation are marked in red. We thank the reviewer for this important comment. We have added an explanation on page 5, lines 122-126.

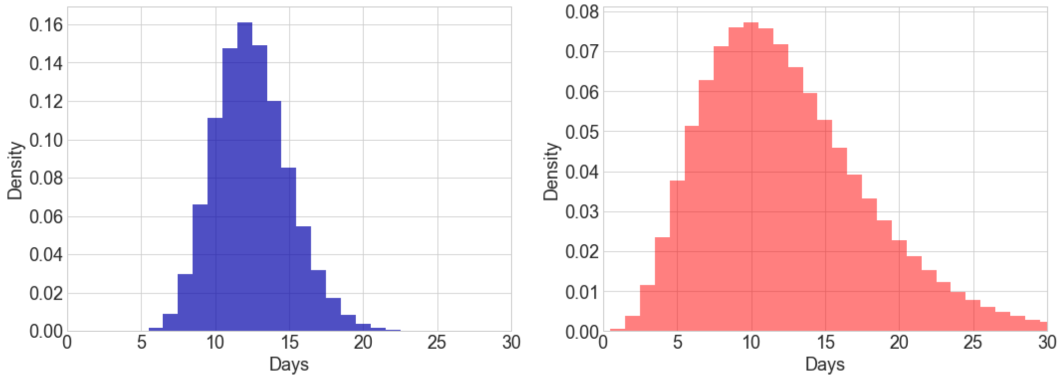

3) Authors may want to explore the role of using aggregated weekly data which may help stabilize the variability in the data series.

3) Thank you for recommending the exploration of using aggregated weekly data. As you rightly pointed out, weekly aggregating data helps reduce the variability and fluctuations associated with daily variations, providing a more stable representation of the underlying trends. Our study primarily focused on utilizing the 10-day smoothed daily time series to address reporting delays and anomalies in the monkeypox incidence data. We chose this approach to maintain interpretability in our dynamic model and have sufficient data to demonstrate how our adaptability model functions. By working with daily data, we can capture the finer-grained patterns and dynamics within shorter time frames. Additionally, using daily data allows us to examine the parameters of the phenomenological model on the same scale as the Rt values, ensuring consistency in the interpretation. However, we acknowledge the potential value of exploring aggregated weekly data as an alternative approach to stabilize the variability and enhance the interpretation of the data. It

could provide a broader perspective and allow a more generalized understanding of the trends over longer periods. We will certainly consider incorporating this suggestion into our future research or as an extension to the current study, as it can offer valuable insights and complement our analysis.

4) The Bayesian sequential approach employed to characterize the temporal uncertainty of the parameters looks interesting. I would like the authors to confirm that the methodology works well with simulated data. Perhaps authors could generate simulated data from the GGM with one step change in "r" and show that the Bayesian approach is useful to infer the correct parameter estimates. How much data is needed to correctly characterize the temporal changes in the parameters?

4) We appreciate the referee's interest in the Bayesian sequential approach employed in our study to characterize the temporal uncertainty of the parameters. We have considered the referee's suggestion and generated simulated data from a negative binomial model like our proposed model for the data using different parameter scenarios. The results demonstrate the effectiveness of the Bayesian approach in inferring the correct parameter estimates. We have included a comprehensive set of synthetic examples in the supplemental material, which covers various scenarios to evaluate the performance of our model. These examples demonstrate the model's ability to accurately capture parameter temporal changes.
Furthermore, we would like to highlight that the forecast performance of our methodology was evaluated in a previous study (Daza-Torres et al., 2022a). In that study, we presented the methodology for the first time and assessed its forecast performance using prediction interval coverage. Specifically, we examined the performance for the total number of confirmed COVID-19 cases and deaths from early-mid March 2020 to February 2021, across 32 Mexican states, forecasting four weeks ahead.
The evaluation metrics employed in the study included the 25% to 75% and 10% to 90% interquartile prediction intervals. The results showed variability among the different states, but in all cases, the forecast performance exceeded 50% and 40% coverage for confirmed cases and deaths, respectively. These findings provide strong evidence that our proposed model accurately characterizes uncertainty, yielding reliable predictions.
In addition to the COVID-19 forecasting application, our sequential Bayesian approach has been applied to other scenarios. For example, we used this approach in an SEIR model with two additional stages for vaccination to project incident cases and deaths in California following the reopening of its economy on June 15, 2021 (Daza-Torres et al., 2022b).

We also recently employed this approach to estimate the COVID-19 positivity rate using SARS-CoV-2 RNA concentrations measured in wastewater, incorporating changes in virus dynamics through an adaptive scheme (Daza-Torres et al., 2023).

- Daza-Torres, Maria L., Marcos A. Capistrán, Antonio Capella, and J. Andrés Christen. "Bayesian sequential data assimilation for COVID-19 forecasting." *Epidemics*, 39 (2022a): 100564.
- Daza–Torres, Maria L., Yury E. García, Alec J. Schmidt, Brad H. Pollock, James Sharpnack, and Miriam Nuño. "The impact of COVID-19 vaccination on California's return to normalcy." *PloS one*, 17, no. 5 (2022b): e0264195.
- Daza-Torres ML, Montesinos-López JC, García YE, Herrera C, Bess CW, Bischel HN, Nuño M. "Bayesian sequential approach to monitor COVID-19 variants through positivity rate from wastewater." *medRxiv* [Preprint]. 2023 Jan 13:2023.01.10.23284365. doi: 10.1101/2023.01.10.23284365. (Accepted in mSystems).

5) Although the goal of the paper is not forecasting the monkeypox epidemics, authors may find interesting that another paper has used related growth models to generate short-term forecasts of the monkeypox epidemics in various areas of the world. The models have yielded competitive forecasts. see: https://bmcmedicine.biomedcentral.com/articles/10.1186/s12916-022-02725-2.

5) Response. As suggested, we have extended our discussion to mention how our results are related to the reference mentioned by the reviewer. We just remark that this reference was not previously included since it was published after our date of submission.

Reviewer 2. The authors of this article focused on estimating the instantaneous reproduction number and growth rates for monkeypox using confirmed cases in Europe. They used a filtering type Bayesian inference to estimate the instantaneous reproduction number and a generalized growth model approximate the epidemic growth rate. The key takeaway from this paper is that a large outbreak of monkey box is low as current propagation of the disease bears a resemblance to stuttering transmission chains. Overall, the article is well-written with insightful results, and the authors make the dissemination of the results easy for the reader to follow.

1) Page 2, Line 93. 1248 should be 1,248.

1) Response. Thanks for noticing, the typo has been corrected.

2) Page 4, Line 122. Citations should be in ascending order.

2) Response. Thanks for noticing, the typo has been corrected.

3) Page 4, Equation 2. I would recommend changing Λ_t to another parameter as it is typically reserved for birth/recruitment rates.

3) Response. As suggested, we have changed the notation to avoid any possible confusion (Ψ_t is used instead of Λ_t)

4) Page 6, Line 182. The t in t-walk should be in math mode.

4) Response. Thanks for noticing, the typo has been corrected.

5) Page 6, Line 182. “The MCMC runs semi-automatic,...” Please clarify this sentence.

5) The phrase 'The MCMC runs semi-automatic' refers to the partial automation of the Markov Chain Monte Carlo (MCMC) process, specifically utilizing the t-walk generic sampler. In this context, 'semi-automatic' means that while the MCMC algorithm performs most of the sampling procedure automatically, the user can adjust certain parameters such as the prior distribution, likelihood, and other tuning parameters based on their expertise and understanding of the problem at hand. This level of customization and control empowers the user to fine-tune the sampling process according to their specific requirements. We have provided additional clarification for this sentence. (See the first paragraph in Page 7)

6) Page 6, Table 1. What is the intuition for using a Beta prior distribution for the deceleration of growth, p?

6) The intuition for using a Beta prior distribution to model the deceleration of growth, p, stems from the properties and characteristics of the Beta distribution. The Beta distribution is suitable for modeling variables with values between 0 and 1, which aligns with the nature of p, representing a probability or proportion.
In this specific case, the initial parameters of the Beta distribution were chosen to provide a nearly uniform probability density across the range of values from 0 to 1. Doing so allows for an unbiased and flexible prior assumption for the deceleration of growth.
To provide a more comprehensive understanding of this intuition's rationale, we have incorporated an additional paragraph in the manuscript that offers detailed explanations regarding the selection of the Beta prior distribution (Page 7). Additionally, a plot illustrating the density of the initial prior distribution of p has been included in the supplementary material. This visual representation provides a clearer insight into the shape

and behavior of the distribution, further supporting the rationale behind the choice of the Beta prior to modeling p. We have employed the same approach for the growth rate parameter, r.

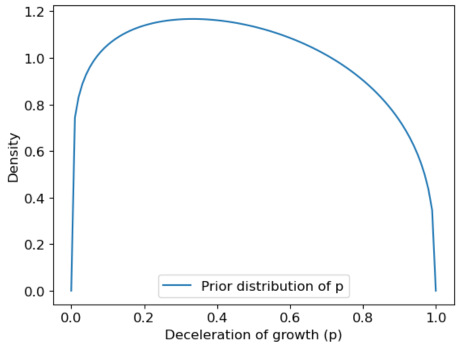

7) Page 6-7, Section 3.1 and 3.2. The authors provide a nice summary of results with respect to the instantaneous reproduction number, growth rates, and subexponential dynamics. I think it would be beneficial to this section if there was some biological discussion on what is influencing these results, e.g. human behavior, disease characteristics, etc.

7) Response. We agree with this comment so we have extended our discussion (see the last paragraph) to improve our explanation of the key factors driving the behavior of the outbreak.

8) Page 7-8, Section 4. Can the authors highlight the robustness of this method? E.g. even though the focus was on monkeypox, how flexible is this methodology to other infectious diseases?

8) In response to the referee's query regarding the robustness of our methodology beyond monkeypox, we would like to emphasize the flexibility and applicability of our approach to various infectious diseases. We have successfully applied this method to COVID-19 data (Daza-Torres et a., 2022a, Daza-Torres et a., 2022b), specifically focusing on cases and hospitalizations, as well as modeling COVID-19 using measurements of SARS-CoV2 detected in wastewater (Daza-Torres et a., 2023).
We would like to highlight that the forecast performance of our methodology was evaluated in a previous study (Daza-Torres et al., 2022a). In that study, we presented the methodology for the first time and assessed its forecast performance using prediction interval coverage. Specifically, we examined the performance for the total number of con-

firmed COVID-19 cases and deaths from early-mid March 2020 to February 2021, across 32 Mexican states, forecasting four weeks ahead. Our findings provide strong evidence that our proposed model accurately characterizes uncertainty, yielding reliable predictions.
To further demonstrate the performance and robustness of our method, we have included a comprehensive set of synthetic examples in the supplemental material of this study. These examples cover various scenarios and accurately evaluate the model's performance, particularly in capturing temporal parameter changes.
We have also added an explanatory paragraph in the discussion section.

– Daza-Torres, Maria L., Marcos A. Capistrán, Antonio Capella, and J. Andrés Christen. "Bayesian sequential data assimilation for COVID-19 forecasting." *Epidemics*, 39 (2022a): 100564.

– Daza–Torres, Maria L., Yury E. García, Alec J. Schmidt, Brad H. Pollock, James Sharpnack, and Miriam Nuño. "The impact of COVID-19 vaccination on California's return to normalcy." *PloS one*, 17, no. 5 (2022b): e0264195.

– Daza-Torres ML, Montesinos-López JC, García YE, Herrera C, Bess CW, Bischel HN, Nuño M. "Bayesian sequential approach to monitor COVID-19 variants through positivity rate from wastewater." *medRxiv* [Preprint]. 2023 Jan 13:2023.01.10.23284365. doi: 10.1101/2023.01.10.23284365. (Accepted in mSystems).

Reviewer 3.

1) Revise line 71 ($Rt < 1$)

1) Response. Thanks for noticing; the typo has been corrected ($Rt = 1$).

2) Risk perception is mentioned, but this variable is not considered in the study, although it could be.

2) Response. It is worth observing that the 2022 mpox outbreak was unexpected and atypical in several aspects. For example, the mpox cases analyzed here were observed in non-endemic regions and mainly affected men who have sex with men (MSM). As mentioned in the discussion, these atypical mpox outbreaks exposed important gaps in understanding the transmission patterns and continuously evolving epidemiological characteristics of the disease. Therefore, instead of proposing a mechanistic epidemic model based on the particular epidemiology of mpox, we use an alternative approach, namely, a flexible phenomenological model to investigate the outbreak. This framework emphasizes the

reproduction of empirical observations but neglects the underlying physical/biological laws. Therefore, we are not considering risk perception as an explicit variable in our approach. We have added this as a limitation of our study in the discussion section.

3) The way of presenting the results figures is striking since they are presented superficially without a description and analysis adequate for understanding.

3) Response. Dear reviewer, we believe the figures are described in enough detail and the other two reviewers did not mention any complaints about the description. Furthermore, we followed the PLOS ONE guidelines about figures: "Be succinct: Avoid lengthy descriptions of methods."

4) line 244 mentions that the estimates reported can help control outbreaks... in what way, by estimating the Rt?

4) Response. We have now extended our discussion to add detail about the usefulness of the estimations presented here. In particular, in the first paragraph of the discussion, we added an explanation about the utility of the estimation of R_t.

5) There is a need to incorporate-discuss estimation strategies from other works with the ones you propose, as it is necessary to visualize the advantages and disadvantages.

5) To address the need for incorporating and discussing estimation strategies from other works alongside our proposed methods, we have now included a comparison between non-adaptive and adaptive estimation approaches in the supplemental material. This addition aims to comprehensively analyze the advantages and disadvantages associated with different estimation strategies.

6) The discussion needs to be strengthened because the manuscript lacks relevance as it is presented.

6) Response. As suggested we have strengthened our discussion by adding a further explanation of the biological aspects driving the behavior of the outbreak. We have also highlighted in more detail the advantages and limitations of our estimation methods.
